# Association between keratin 8 expression and immune-related pneumonitis: a case-control study in lung adenocarcinoma patients

**DOI:** 10.3389/fimmu.2025.1573943

**Published:** 2025-07-24

**Authors:** Xuena Wang, Yang Yang, Hongxia Guo, Xu Li, Yue Sun, Qi Mei, Yuhui Ma

**Affiliations:** ^1^ Shanxi Bethune Hospital, Shanxi Academy of Medical Sciences, Tongji Shanxi Hospital, Third Hospital of Shanxi Medical University, Taiyuan, Shanxi, China; ^2^ Department of Ophthalmology, Renmin Hospital of Wuhan University, Wuhan, Hubei, China; ^3^ Department of Endoscopy, Guangdong Provincial People’s Hospital (Guangdong Academy of Medical Sciences), Southern Medical University, Guangzhou, China; ^4^ Department of Oncology, Tongji Hospital, Tongji Medical College, Huazhong University of Science and Technology, Wuhan, Hubei, China

**Keywords:** immune-related pneumonitis, keratin 8, lung adenocarcinoma, case-control, lung cancer

## Abstract

**Background:**

Immune-related pneumonitis (IRP) is a serious adverse event observed in lung adenocarcinoma patients undergoing immunotherapy. Previous studies have revealed that high Keratin 8 (KRT8) expression is associated with poor prognosis in these patients. However, the potential link between KRT8 expression levels and the risk of developing IRP remains unclear. This study aims to explore the correlation between KRT8 expression and IRP risk in lung adenocarcinoma patients receiving immunotherapy.

**Methods:**

A case-control study was conducted involving 36 lung adenocarcinoma patients (12 IRP cases and 24 age- and sex-matched controls without IRP) receiving immunotherapy. Tumor tissue samples were analyzed for KRT8 expression using immunohistochemistry. A multivariate logistic regression analysis was performed to evaluate the relationship between KRT8 expression and IRP risk.

**Results:**

KRT8 expression was significantly higher in the IRP group compared to controls (12.9 ± 8.2% vs 5.6 ± 5.2%, adjusted *P* = 0.03). Multivariate analysis revealed that each percentage increase in KRT8 expression was associated with a 32% increased risk of developing IRP (OR = 1.32, 95% CI: 1.09-1.61, *P* = 0.005). Compared to the lowest tertile, the moderate KRT8 expression tertile showed no significant association with IRP risk, while the highest tertile demonstrated a significant 14-fold increased risk of developing IRP.

**Conclusion:**

Elevated KRT8 expression in tumor tissues is significantly associated with increased IRP risk in lung adenocarcinoma patients receiving immunotherapy. These exploratory, hypothesis-generating findings suggest that KRT8 expression may serve as a potential biomarker for predicting IRP development, though validation in larger cohorts is needed.

## Introduction

Lung cancer remains the leading cause of cancer-related mortality worldwide, with lung adenocarcinoma being the most prevalent type of non-small cell lung cancer (NSCLC) (1). In recent years, the advent of immunotherapy, particularly the use of immune checkpoint inhibitors (ICIs), has transformed the treatment landscape for lung cancer (2). ICIs, primarily targeting programmed death receptor-1 (PD-1) and its ligand PD-L1, have significantly improved overall survival rates for lung cancer patients (2). However, as the application of immunotherapy expands, the recognition and management of associated toxicities, particularly immune-related adverse events (irAEs), have become critically important. Among these irAEs, immune-related pneumonitis (IRP) stands out for its potential severity and the challenges it presents in clinical management.

IRP is a common adverse effect observed in patients receiving ICIs (3). Retrospective epidemiological studies have reported an incidence of IRP ranging from 3.5% to 19%, accounting for approximately 35% of deaths associated with anti-PD-1/PD-L1 therapies (4–6). The pathophysiology of IRP is complex and not fully understood, intertwining immune system activation with underlying pulmonary pathology (4). Clinical manifestations of IRP can vary significantly, ranging from mild symptoms such as cough and dyspnea to severe respiratory failure, which may require intensive care and corticosteroid treatment (7). This variability in clinical presentation presents significant challenges for the diagnosis and management of IRP.

Identifying predictive markers for IRP could significantly enhance clinical practice by facilitating proactive monitoring and management of at-risk patients. While previous studies have explored various potential biomarkers, including peripheral blood markers and radiographic imaging patterns (8, 9), no consensus has been established regarding reliable predictors for pneumonitis development. This knowledge gap underscores the urgent need for continued research to improve prediction and prevention strategies for IRP, thereby maximizing the therapeutic benefits of ICIs while minimizing associated risks.

Keratin 8 (KRT8), an intermediate filament protein, plays a critical role in protecting against oxidative stress, maintaining mitochondrial homeostasis, and preventing apoptosis (10–12). Recent evidence suggests a potential link between KRT8 and the progression of various tumors, highlighting its involvement in key processes such as cell migration, adhesion, and drug resistance (13–15). Notably, alterations in KRT8 expression have been observed across multiple cancer types, including lung adenocarcinoma, indicating its potential utility as a biomarker for disease progression and treatment response (16–19). However, the relationship between KRT8 expression and IRP, particularly in the context of lung adenocarcinoma immunotherapy, has not been extensively explored. This current study aims to examine KRT8 expression levels in lung adenocarcinoma patients undergoing immunotherapy, thereby providing a valuable tool for stratifying patients at heightened risk of developing IRP.

## Materials and methods

### Study design and patient selection

This research was a retrospective study carried out at the Tumor Center of Shanxi Bethune Hospital. We screened the medical records of lung adenocarcinoma patients who were hospitalized and received immune checkpoint inhibitor therapy between January 2022 and December 2023. At this institution, all patients undergo comprehensive baseline lung function assessments prior to immunotherapy initiation, and only patients with normal lung function are eligible for treatment. Additionally, immunotherapy is exclusively administered to patients with PD-L1 expression >1%. Among patients meeting these criteria, those who developed immune-related pneumonitis, confirmed by radiographic imaging, were identified. A total of 12 cases of immune-related pneumonitis were included in the study. For comparison, we selected lung adenocarcinoma patients treated with immune checkpoint inhibitors within the same timeframe, who did not develop immune-related pneumonitis during their treatment course. It should be noted that the control group selection was based specifically on the absence of IRP, and patients were not systematically screened for the presence of other irAEs. These control patients were matched with the cases on a 1:2 basis, according to age and sex, resulting in a control group of 24 patients.

### Ethical approval

The study protocol was reviewed and approved by the Ethics Committee of Shanxi Bethune Hospital, ensuring compliance with the ethical standards of the Declaration of Helsinki. Written informed consent was obtained from all participants or their legal guardians before inclusion in the study.

### KRT8 expression measurement

Formalin-fixed, paraffin-embedded human lung tumour tissues were sectioned and processed for KRT8 immunohistochemical (IHC) staining. Sections were deparaffinised by sequential immersion in three separate dewaxing solutions for 15 minutes each, followed by rehydration in graded concentrations of anhydrous ethanol (100%, 85%, and 75%, 5 minutes each), and then rinsed in distilled water. Antigen retrieval was accomplished by immersing the slides in citrate buffer and heating in a microwave oven for 20 minutes, after which slides were cooled to room temperature and washed three times in phosphate buffered saline (PBS) for 5 minutes each.

Endogenous peroxidase activity was blocked by incubating the sections in 3% hydrogen peroxide, protected from light at room temperature for 25 minutes, followed by three further washes with PBS, each for 5 minutes. Non-specific binding was minimised by incubating with bovine serum albumin for 20 minutes at room temperature. The sections were then incubated overnight at 4°C with primary antibody against KRT8 (Proteintech, 17514-1-AP; 1:200 dilution). After three washes in PBS, slides were incubated with HRP-conjugated goat anti-rabbit secondary antibody (Servicebio, GB22303; 1:100 dilution) at 37°C for 30 minutes, followed by three additional PBS washes.

Visualisation was performed with freshly prepared Diaminobenzidine (DAB) substrate solution (Zhongshan Golden Bridge, ZL1-9018; 1:20 dilution). The reaction was monitored microscopically and terminated by rinsing the slides in distilled water when a brown-yellow (positive) signal was observed. Nuclear counterstaining was carried out with haematoxylin for 3 minutes, followed by bluing in tap water and rinsing in running water. Slides were dehydrated in a graded alcohol series (75%, 85%, 95%, and 100%) and xylene, each for 10 minutes, and subsequently mounted using neutral resin. Positive KRT8 expression was identified as brown-yellow staining predominantly in the cytoplasm, while nuclei were counterstained blue with haematoxylin. Two pathologists, blinded to clinical outcomes, confirmed tissue adequacy and staining quality.

**Figure 1:**
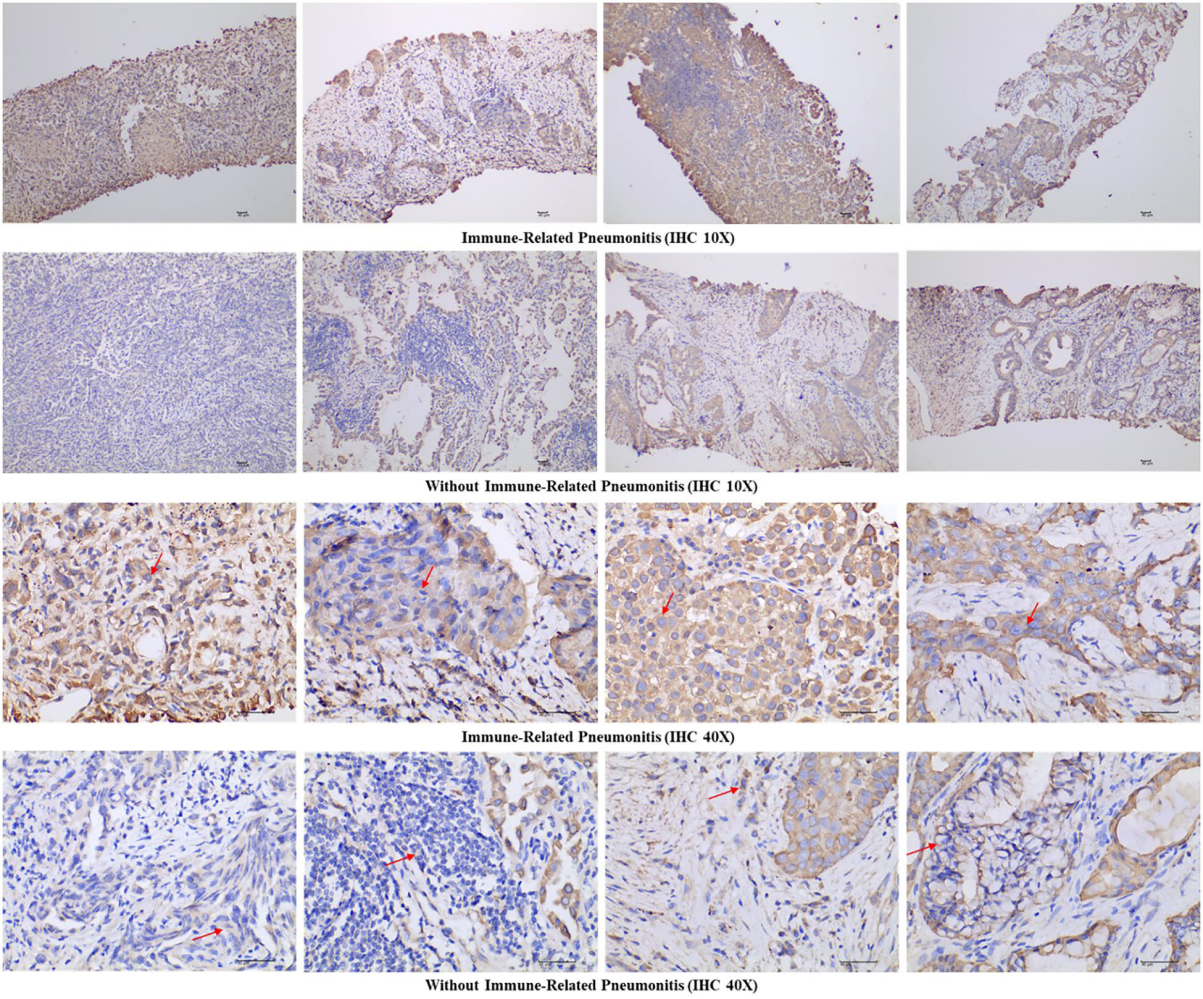
Representative immunohistochemical images of Keratin 8 staining in both immune-related pneumonitis cases and controls at 10× and 40× magnification. Arrows indicate cytoplasmic Keratin 8 expression.

Following IHC staining, tissue sections were analysed using a digital three-lens microscope system (BA400Digital, McAudi Industrial Group Co., Ltd.). For each specimen, the entire tissue area was observed at low magnification, followed by acquisition of representative images at 100× and 400× magnification (**Figure 1**). Quantification of KRT8 expression was conducted using the Halo image analysis system (Indica Labs, U.S.A., model: 101-WL-HALO-1). The system calculated the percentage of DAB-positive tissue area (% DAB Positive Tissue) for each 400× image, representing the proportion of tissue exhibiting positive KRT8 expression relative to the total tissue area analysed. The selection of percent positive area was based on several key considerations: (1) the proportion of phenotypically altered cells provides more reliable prognostic information by directly reflecting tumor biological behavior (20); (2) area-based measurements are less susceptible to pre-analytical variables such as fixation time and antigen retrieval conditions that can affect staining intensity; and (3) automated digital pathology platforms provide enhanced reproducibility across different laboratories and scanning conditions. This automated quantification approach (range: 0-100%) eliminates subjective interpretation and provides standardized, reproducible measurements.

### Demographic and clinical data collection

For each patient, demographic data (age, sex), clinical characteristics (smoking history, other lung diseases, number of comorbidities), and laboratory indicators (IL-6, IL-8, IL-10, white blood cell count, neutrophil, lymphocyte, eosinophil, hemoglobin, and platelet levels) were retrieved from electronic health records maintained by the hospital.

### Statistical analysis

Statistical analysis was performed using R 4.3.2. Statistical significance for differences in categorical variables was calculated using the chi-square test or Fisher’s exact test, as appropriate. For continuous variables, the Mann–Whitney U test was utilized. To address potential Type I error inflation due to multiple comparisons, the Benjamini-Hochberg false discovery rate (FDR) correction was applied to all baseline characteristic comparisons. Both uncorrected and FDR-adjusted *P*-values are reported. We employed conditional logistic regression, Least Absolute Shrinkage and Selection Operator (LASSO) regression and Bayesian logistic regression analysis (21, 22). For conditional logistic regression, variables with significant differences (*P* < 0.1) in baseline characteristics were included as covariates. To assess model stability and reliability, we performed bootstrap validation using 1000 resampling iterations of the conditional logistic regression model. Bootstrap confidence intervals were calculated using the percentile method. Model discrimination was assessed using the concordance index (C-index). For bootstrap validation, only models with reasonable parameter estimates (coefficient < 3, OR between 0.1-10) were included in the final analysis to ensure statistical stability. Additionally, leave-one-out cross-validation was conducted to further evaluate model performance and stability. For the LASSO analysis, we included all available clinical and laboratory variables (age, sex, IL-6, IL-8, IL-10, KRT8 expression, smoking history, other lung diseases, number of comorbidities, white blood cell count, neutrophils, lymphocytes, eosinophils, hemoglobin, and platelets) in a penalized regression model. LASSO regression was performed using the glmnet package in R, with 10-fold cross-validation to determine optimal regularization parameters (lambda.min and lambda.1se). Furthermore, we employed Bayesian logistic regression using the arm package in R to provide more robust parameter estimation while adjusting for potential confounders and smoking history. Odds ratios (ORs) and their corresponding 95% confidence intervals (CIs) were calculated. A two-sided *P*-value < 0.05 was considered statistically significant.

To explore potential non-linear relationships between KRT8 expression and IRP risk, additional Bayesian logistic regression analyses were performed to explore potential threshold effects by modeling KRT8 expression as categorical variables using tertiles. Bayesian analysis was conducted using weakly informative priors. ORs and their corresponding 95% credible interval (CrIs) were calculated, and posterior probabilities *P* (OR>1) were calculated to assess the probability that ORs exceeded unity. All models were adjusted for the same covariates as in the primary analysis.

## Results

This study included 36 lung adenocarcinoma patients, comprising 24 patients without IRP and 12 patients who developed IRP.

### Baseline characteristics

There were no significant differences between the two groups in terms of age (without IRP: 66.1 ± 6.2 years vs IRP: 66.9 ± 7.1 years, *P* = 0.719) or gender distribution (male proportion 75.0% vs 83.3%, *P* = 0.887) (**Table 1**). Smoking history (87.5% vs 100%, *P* = 0.522) and other pulmonary diseases (41.7% vs 33.3%, *P* = 0.904) were also comparable between groups. Although not reaching statistical significance (*P* = 0.077), the IRP group appeared to have a higher comorbidity burden, with 58.3% of patients having one or more comorbidities compared to 20.9% in the non-IRP group (**Table 1**).

**Table 1:** Participant characteristics (n=36) ^a^.

Characteristics	Without IRP (n=24)	IRP (n=12)	*P* value	FDR adjusted *P* value
Age (years)	66.1 ± 6.2	66.9 ± 7.1	0.719	0.962
Sex (male, %)	18.0 (75.0)	10.0 (83.3)	0.887	0.962
IL-6 (pg/mL)	15.3 ± 18.7	28.4 ± 45.0	0.224	0.672
IL-8 (pg/mL)	5.8 ± 4.9	8.9 ± 5.5	0.101	0.379
IL-10 (pg/mL)	3.1 ± 2.0	4.6 ± 2.2	0.042	0.315
KRT8 (%)	5.6 ± 5.2	12.9 ± 8.2	0.002	0.030
Smoke history (yes, %)	21.0 (87.5)	12.0 (100.0)	0.522	0.962
Other lung disease (yes, %)	10.0 (41.7)	4.0 (33.3)	0.904	0.962
Number of comorbidities (n, %)			0.077	0.379
0	19.0 (79.2)	5.0 (41.7)		
1	4.0 (16.7)	6.0 (50.0)		
2	1.0 (4.2)	1.0 (8.3)		
WBC (10^9^/L)	8.3 ± 2.9	8.2 ± 2.4	0.962	0.962
Neutrophil (10^9^/L)	5.8 ± 2.5	5.7 ± 1.8	0.823	0.962
Lymphocyte (10^9^/L)	1.7 ± 0.6	1.7 ± 0.8	0.939	0.962
Eosinophil (10^9^/L)	0.2 ± 0.3	0.1 ± 0.1	0.304	0.753
Hemoglobin (10^9^/L)	125.8 ± 20.5	128.2 ± 21.6	0.749	0.962
Platelet (10^9^/L)	285.4 ± 95.8	317.3 ± 117.8	0.39	0.836

IRP, immune-related pneumonitis; FDR, false discovery rate; IL, interleukin; KRT8, keratin 8; WBC, white blood cell.

a Continuous variables are expressed as and categorical variables are expressed as percentages.

**Figure 2:**
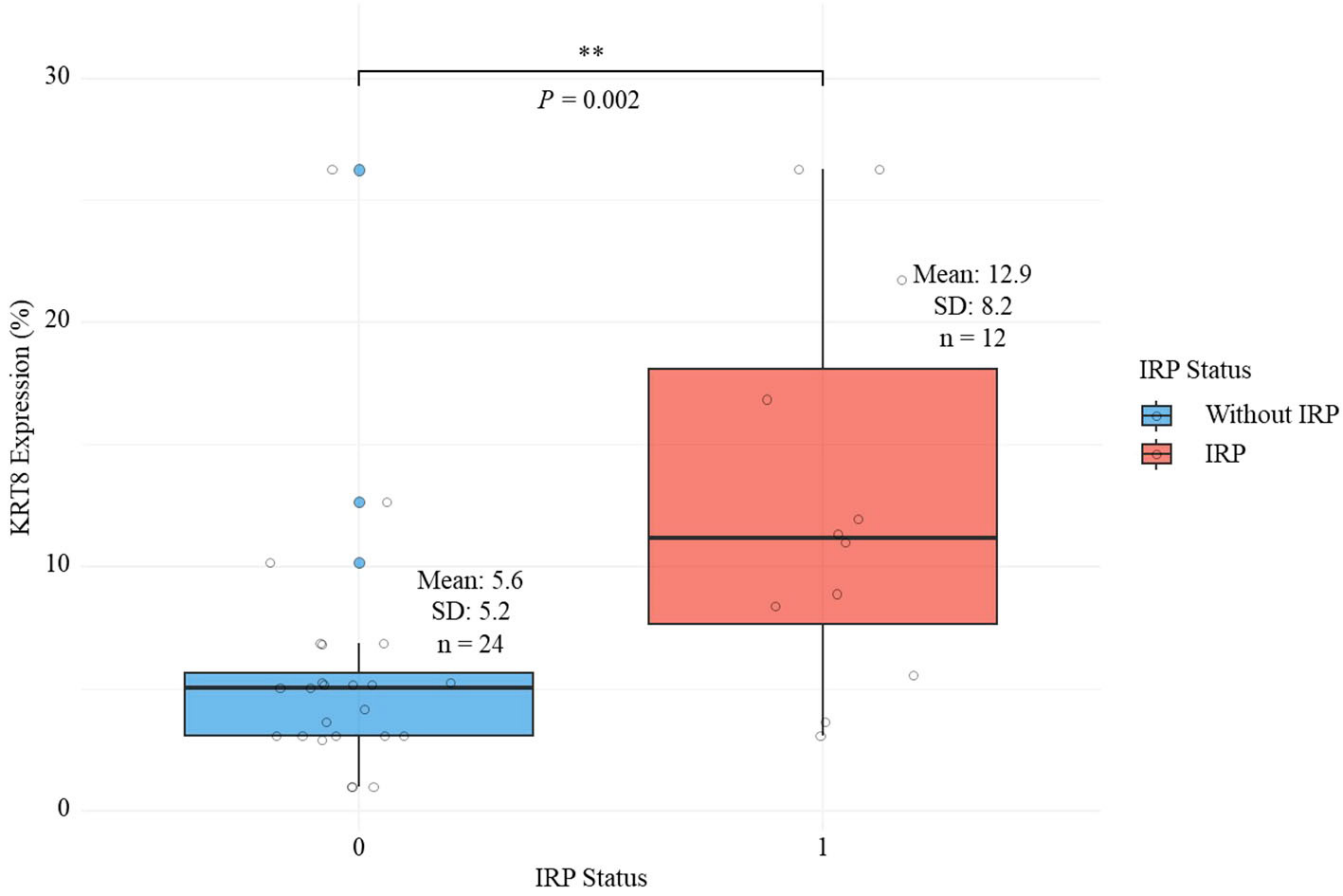
Comparison of keratin 8 expression levels between lung adenocarcinoma patients with and without immune-related pneumonitis.

Patients in the IRP group demonstrated significantly higher IL-10 levels compared to the non-IRP group (4.6 ± 2.2 pg/mL vs 3.1 ± 2.0 pg/mL, *P* = 0.042) (**Table 1**). Although IL-6 (28.4 ± 45.0 vs 15.3 ± 18.7 pg/mL, *P* = 0.224) and IL-8 (8.9 ± 5.5 vs 5.8 ± 4.9 pg/mL, *P* = 0.101) levels were elevated in the IRP group, these differences did not reach statistical significance (**Table 1**). Most notably, KRT8 expression levels were significantly higher in the IRP group compared to the non-IRP group (12.9 ± 8.2% vs 5.6 ± 5.2%, *P* = 0.002) (**Table 1**; **Figure 2**). No significant differences were observed between groups in white blood cell count (8.2 ± 2.4 vs 8.3 ± 2.9 ×10^9^/L, *P* = 0.962), neutrophils (5.7 ± 1.8 vs 5.8 ± 2.5 ×10^9^/L, *P* = 0.823), lymphocytes (1.7 ± 0.8 vs 1.7 ± 0.6 ×10^9^/L, *P* = 0.939), eosinophils (0.1 ± 0.1 vs 0.2 ± 0.3 ×10^9^/L, *P* = 0.304), hemoglobin (128.2 ± 21.6 vs 125.8 ± 20.5 g/L, *P* = 0.749), and platelets (317.3 ± 117.8 vs 285.4 ± 95.8 ×10^9^/L, *P* = 0.39) (**Table 1**).

Following Benjamini-Hochberg FDR correction for multiple testing across all baseline variables, KRT8 expression remained significantly associated with IRP development (FDR-adjusted *P* = 0.03), while IL-10 levels, which showed significance in uncorrected analysis (*P* = 0.042), did not maintain significance after correction (FDR-adjusted *P* = 0.315). All other variables remained non-significant after FDR adjustment (**Table 1**).

### Association between KRT8 and IRP

Univariate and multivariate logistic regression analyses were performed to investigate the association between KRT8 expression and IRP. In the univariate conditional logistic analysis, KRT8 expression showed a significant positive correlation with the occurrence of IRP (OR = 1.15, 95% CI: 1.01-1.32, *P* = 0.038) (**Table 2**). The multivariate conditional logistic analysis, which adjusted for potential confounding factors, further confirmed this association. KRT8 expression remained significantly associated with immune-related pneumonia (OR = 1.21, 95% CI: 1.01-1.45, *P* = 0.042) (**Table 2**). Other factors included in the multivariate conditional logistic analysis, such as IL-10 levels (OR = 1.69, 95% CI: 0.84-3.43, *P* = 0.143) and the presence of comorbidities (single comorbidity: OR = 5.62, 95% CI: 0.28-114.2, *P* = 0.261; two comorbidities: OR = 0.16, 95% CI: 0.001-35.2, *P* = 0.507), did not show statistically significant associations with immune-related pneumonia (**Table 2**). Bootstrap validation revealed a convergence rate of 53.6% (536/1000 iterations), with a mean KRT8 odds ratio of 1.95 (95% CI: 0.94-6.87). The model demonstrated excellent discriminative ability with a mean C-index of 0.91 (95% CI: 0.69-1.00). Leave-one-out cross-validation showed 100% model convergence (36/36 successful fits) with a mean KRT8 coefficient of 0.23 (standard deviation: 0.15) and mean odds ratio of 1.28, closely approximating the primary analysis results.

**Table 2:** Univariate and multivariate analysis of the association between KRT8 and immune-related pneumonia.

Variable	Conditional logistic regression		Conditional logistic regression		Bayesian logistic regression	
	OR (95%CI)	*P* value	OR (95%CI)	*P* value	OR (95%CI)	*P* value
KRT8 (%)	1.15 (1.01, 1.32)	0.038	1.21 (1.01, 1.45)	0.042	1.32 (1.09-1.61)	0.005
IL-10 (pg/mL)	–	–	1.69 (0.84, 3.43)	0.143	1.70 (1.07-2.72)	0.026
Number of comorbidities=1	–	–	5.62 (0.28, 114.2)	0.261	4.49 (0.60-33.56)	0.143
Number of comorbidities=2	–	–	0.16 (0.001, 35.2)	0.507	1.22 (0.02-69.41)	0.924
Smoking history (yes)	–	–	–	–	64.87 (0.87-4859.45)	0.058

OR, odds ratio; CI, confidence interval; KRT8, keratin 8, IL, interleukin.

**Figure 3:**
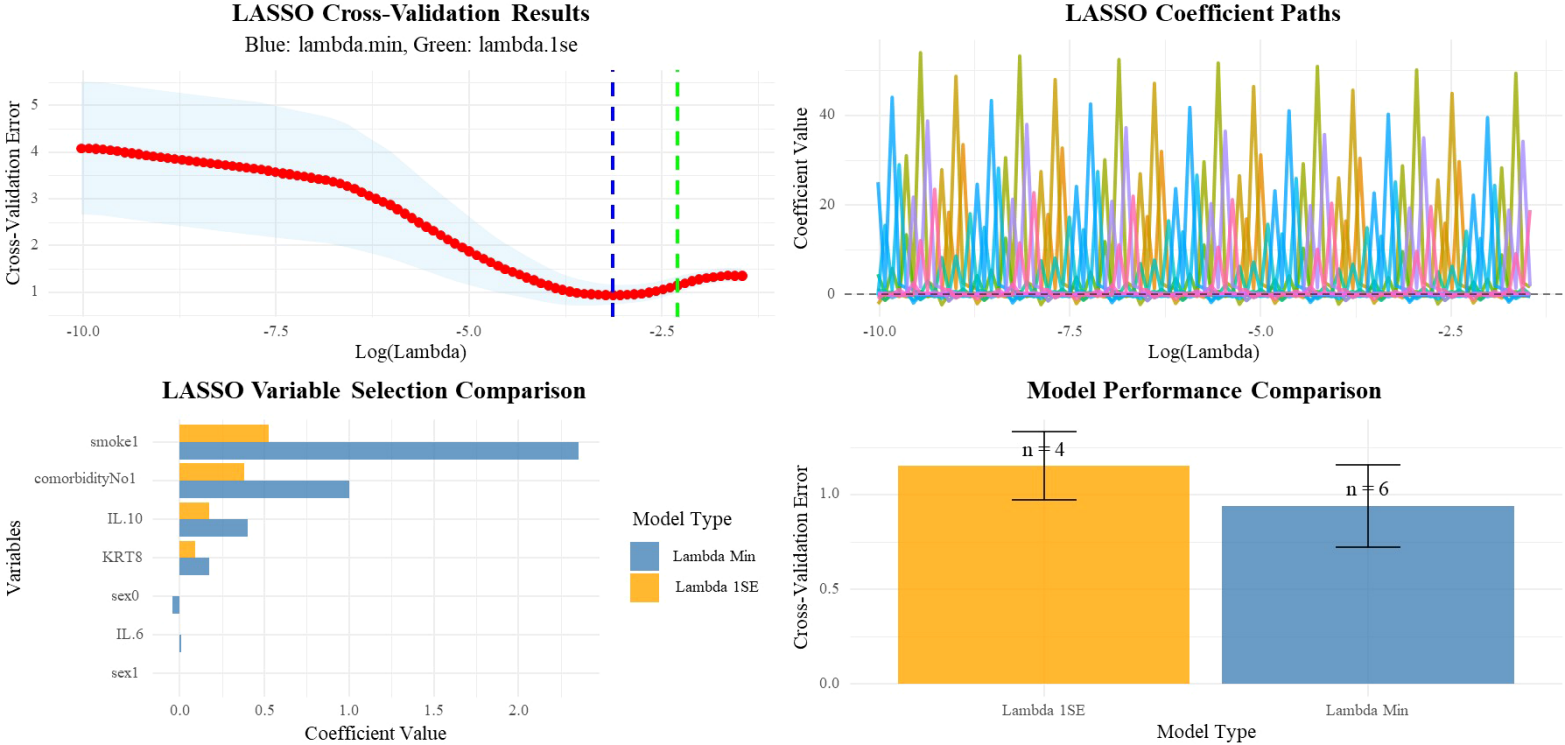
LASSO regularization analysis of risk factors associated with immune-related pneumonia.

To validate the results, we further performed LASSO regression analysis including all available clinical and laboratory variables (**Figure 3**). The LASSO analysis consistently selected KRT8 as a significant predictor across both optimal lambda values (lambda.min: coefficient = 0.176; lambda.1se: coefficient = 0.092), confirming the robustness of the KRT8-IRP association. Other variables consistently selected by LASSO included IL-10 (coefficients: 0.402 and 0.175), smoking history (coefficients: 2.357 and 0.527), and comorbidity count (coefficients: 1.003 and 0.381), supporting their biological relevance and inclusion in our multivariate model. The multivariate Bayesian logistic analysis, which adjusted for potential confounding factors including smoking history, further confirmed this association. KRT8 expression remained significantly associated with immune-related pneumonitis (OR = 1.32, 95% CI: 1.09-1.61, *P* = 0.005) (**Table 2**). Additionally, IL-10 levels showed a significant association with IRP risk (OR = 1.70, 95% CI: 1.07-2.72, *P* = 0.026), while smoking history and the presence of comorbidities did not show statistically significant associations with immune-related pneumonitis (**Table 2**).

### Threshold effect analysis of KRT8 expression

To explore potential non-linear relationships between KRT8 expression and IRP risk, we performed additional analyses modeling KRT8 as categorical variables using tertiles. The tertile cutoffs were: low (0.99%-3.65%), moderate (4.17%-6.86%), and high (8.37%-26.3%) KRT8 expression levels. Bayesian logistic regression analysis revealed a clear threshold effect (**Table 3**). Patients in the high KRT8 expression tertile demonstrated substantially increased odds of developing IRP (OR = 14.04, 95% CrI: 2.55-91.70, *P* (OR>1) = 0.999), while the moderate tertile showed no significant association with IRP risk (OR = 0.38, 95% CrI: 0.03-3.18, *P* (OR>1) = 0.203). This pattern remained consistent across all adjusted models (Model 2: OR = 126.78, 95% CrI: 9.65-2275.36; Model 3: OR = 113.15, 95% CrI: 9.65-1965.68).

**Table 3:** Bayesian logistic analysis of the association between tertiles of KRT8 and immune-related pneumonia.

Variable	Model 1		Model 2		Model 3	
	OR (95%CrI)	*P* (OR>1)	OR (95%CrI)	*P* (OR>1)	OR (95%CrI)	*P* (OR>1)
KRT8 Tertiles						
Low (0.99%-3.65%)	1 (Reference)	–	1 (Reference)	–	1 (Reference)	–
Moderate (4.17%-6.86%)	0.38 (0.03-3.18)	0.203	0.56 (0.04-7.92)	0.345	0.47 (0.02-6.65)	0.296
High (8.37%-26.3%)	14.04 (2.55-91.70)	0.999	126.78 (9.65-2275.36)	1.000	113.15 (9.65-1965.68)	1.000
IL-10 (pg/mL)	–	–	2.05 (1.21-3.70)	0.997	2.21 (1.22-4.20)	0.996
Number of comorbidities						
0	–	–	1 (Reference)	–	1 (Reference)	–
1	–	–	28.30 (2.73-405.92)	0.998	21.37 (1.82-318.33)	0.992
2	–	–	1.13 (0.03-45.60)	0.522	1.36 (0.03-101.70)	0.543
Smoking history						
No	–	–	–	–	1 (Reference)	–
Yes	–	–	–	–	12.44 (0.45-470.19)	0.927

OR, odds ratio; CrI, credible interval; *P* (OR>1), posterior probability that odds ratio is greater than 1; KRT8, keratin 8, IL, interleukin.

## Discussion

This study demonstrated that elevated KRT8 expression serves as a significant biomarker for IRP development in lung adenocarcinoma patients receiving immunotherapy. KRT8 expression levels were substantially higher in patients who developed IRP and remained independently associated with IRP risk after multivariate adjustment, with each percentage increase conferring 32% increased odds of developing pneumonitis. Notably, a threshold effect was observed, where patients in the highest KRT8 expression tertile showed dramatically increased IRP risk compared to lower expression levels. These association maintained statistical significance after multiple testing correction, providing initial but valuable evidence supporting the hypothesis that KRT8 may serve as a predictive biomarker for IRP, warranting investigation in larger cohorts. While previous studies have investigated various biomarkers for IRP prediction, to our knowledge, this is the first study to identify KRT8 as a potential predictive marker.

KRT8 is part of the keratin gene family, which encodes for intermediate filament proteins that are crucial for the mechanical stability and integrity of epithelial cells (23, 24). While the role of keratins is well-studied in the context of epithelial integrity and disease (23), their involvement in immune responses, particularly in the context of immunotherapy-induced adverse events, is less understood. Our findings are in line with studies suggesting that alterations in cytoskeletal proteins, such as keratins, may influence cancer progression and the tumor microenvironment’s response to immunotherapy (25). The elevated expression of KRT8 in patients who developed IRP could reflect underlying changes in epithelial cell stability and immunity (23, 26). This suggests that KRT8 might not just be a bystander in the process but could actively participate in the pathological mechanism leading to IRP when the immune system is modulated by therapy. Besides, the correlation between increased expression of certain keratins and heightened inflammatory responses has been documented in other contexts (27, 28). For instance, keratin mutations in epithelial tissues have been shown to lead to chronic inflammation and fibrosis (28–32), conditions that share pathological features with IRP. Our study adds to the body of evidence by quantitatively linking KRT8 expression with IRP occurrence in a clinically relevant setting.

The exact mechanism by which elevated KRT8 expression leads to heightened susceptibility to IRP remains unknown and requires further investigation. Based on existing literature, we can propose several hypothetical mechanisms that warrant experimental validation. First hypothesis: increased KRT8 expression may potentially destabilize epithelial cell junctions, possibly leading to enhanced permeability and susceptibility to infiltration by immune cells (33). This hypothetical mechanism could potentially set the stage for an exaggerated inflammatory response when challenged by ICIs, though this remains to be experimentally demonstrated. Second hypothesis: KRT8 might potentially interact with signaling pathways linked to inflammation and immune regulation. For example, some studies have suggested KRT8’s involvement in NF-κB pathway modulation, a central mediator of inflammatory responses (34). We hypothesize that dysregulation of NF-κB signaling could potentially prime the lung epithelium for an overactive response to immunotherapy, culminating in IRP (34), though direct evidence for this mechanism in the context of IRP is lacking. Third hypothesis: changes in KRT8 expression may potentially influence the tumor microenvironment, possibly affecting the recruitment and activation of immune cells (35). This hypothetical altered immune landscape could predispose patients to IRP by creating a pro-inflammatory environment that responds excessively to ICIs. However, all these proposed mechanisms remain speculative and require rigorous experimental validation through *in vitro* and *in vivo* studies.

Recent investigations have explored various biomarkers for IRP prediction, focusing primarily on peripheral inflammatory markers, hematological indices, and radiographic patterns. A multicenter retrospective study of 107 advanced lung cancer patients with checkpoint inhibitor-related pneumonitis (CIP) found elevated IL-6 and CRP levels at the time of ICI rechallenge were significantly associated with higher CIP recurrence rates (36), which suggests that patients with elevated IL-6 and CRP may be in an inflammatory state during rechallenge, making them more susceptible to CIP recurrence. Similarly, a retrospective, observational study examining 87 lung cancer patients with CIP and 87 matched controls demonstrated that elevated levels of inflammatory cytokines IL-6 and IL-10, increased neutrophil-to-lymphocyte ratio and platelet-to-lymphocyte ratio, and elevated lactate dehydrogenase were significantly associated with CIP occurrence, while decreased absolute lymphocyte count and reduced albumin levels also correlated with pneumonitis development (37). Radiographic patterns have also emerged as important prognostic indicators. Early-onset IRP characteristically exhibits organizing pneumonia patterns associated with poor prognosis, whereas late-onset cases typically demonstrate non-specific interstitial pneumonia patterns (38). Although these biomarkers have contributed to improved understanding and prediction of IRP, they may not fully capture the complexity of disease pathogenesis. KRT8, as a tumor tissue biomarker, may capture unique aspects of epithelial biology and immune susceptibility not reflected in circulating markers or radiographic changes. Integrating KRT8 with established biomarkers could potentially enhance risk stratification for IRP, supporting more precise and personalized patient management during immunotherapy.

While our findings are promising, several important limitations must be acknowledged. First, the main limitation of this work was the small sample size, which impacts the statistical power and, consequently, the generalizability of our conclusions. *Post-hoc* power analysis suggests moderate power (53~77%) to detect the observed difference in KRT8 expression, but insufficient power for small effect sizes. The bootstrap validation showing model instability with only 53.6% convergence rate across resampling iterations. Bootstrap analysis revealed substantial statistical bias, indicating potential imprecision in parameter estimates under small sample conditions. To enhance the reliability of our findings despite these limitations, we employed additional validation approaches. Leave-one-out cross-validation demonstrated relative stability with 100% model convergence and consistent results. Furthermore, to address potential concerns about variable selection bias in small samples, we supplemented our conventional regression analysis with LASSO regression, which confirmed the robustness of the KRT8-IRP association through penalized variable selection. While these validation methods strengthen confidence in our results, the findings should still be interpreted as preliminary evidence requiring validation in larger, multi-center studies. Second, although our institutional protocols standardized baseline pulmonary function (normal for all patients) and PD-L1 status (>1% for all patients), formal matching for these variables was not performed. While this standardization effectively controlled for these confounding factors, and our Bayesian analysis adjusted for smoking history and other potential confounders, future studies would benefit from explicit matching criteria that include comprehensive risk factors such as smoking intensity, specific baseline pulmonary function parameters, exact PD-L1 expression levels, and immunotherapy regimen details. Third, our control group definition focused exclusively on the absence of IRP without systematic exclusion of patients who may have experienced other irAEs during immunotherapy. This could potentially introduce confounding factors, as patients with other irAEs may have different baseline immune activation patterns that could influence biomarker expression. Future studies should consider implementing more stringent control group criteria that exclude all patients with documented irAEs to ensure cleaner group comparisons and more definitive conclusions about the specific association between KRT8 expression and IRP risk. Fourth, our single-center, retrospective study design without an independent validation cohort substantially limits the clinical utility and generalizability of KRT8 as a predictive biomarker for IRP. The absence of external validation prevents us from establishing the reproducibility and robustness of the KRT8-IRP association across different patient populations, institutional practices, and geographic regions. Fifth, our study design does not allow for causal inferences. Prospective studies investigating the temporal dynamics of KRT8 expression relative to immunotherapy administration and IRP development are warranted. Last, our study establishes a statistical association between KRT8 expression and IRP risk but does not provide mechanistic insights. The proposed mechanisms discussed are purely hypothetical and based on extrapolation from other disease contexts. Future experimental studies are critically needed to: (1) validate the proposed mechanistic pathways linking KRT8 to inflammatory responses in lung epithelium; (2) demonstrate causality rather than mere association; (3) investigate the temporal relationship between KRT8 expression changes and immune activation; and (4) elucidate whether KRT8 directly contributes to IRP pathogenesis or serves merely as a biomarker reflecting underlying pathological processes.

In conclusion, our exploratory study provides preliminary evidence that KRT8 expression may serve as a predictive biomarker for IRP in lung adenocarcinoma patients treated with immunotherapy, while the underlying biological mechanisms remain to be elucidated. While these hypothesis-generating findings are promising, rigorous validation in larger, multicenter, prospective cohorts with standardized protocols is essential before any clinical implementation can be considered.

## Data Availability

The raw data supporting the conclusions of this article will be made available by the authors, without undue reservation.
